# Supplementary material for: Sniffing speeds up chemical detection by controlling air-flows near sensors
Source: Nat Commun. 2021 Feb 23;12:1232. doi: 10.1038/s41467-021-21405-y (PMC7902652; doi:10.1038/s41467-021-21405-y)
Supplement: Supplementary file 1 — Supplementary Information [file 41467_2021_21405_MOESM1_ESM.pdf]

## 1 Supplementary Methods

**Predicting sensor current oscillations for flows at different Womeresly number** Here, we derive the rate of molecules accruing on the sensor. Our goal is to derive a relationship for the maximum possible number of molecules that can arrive on the sensor by diffusion. This involves estimating the volume of molecules that are in the vicinity of the sensor at each time point and are traveling slow enough to have the potential to diffuse onto the surface.

Since diffusion is a random process, many of the molecules do not land on the surface of the sensor. The total number of molecules $N_{\text{S}}$ which land on the sensor may be written as the product of the collection efficiency $\eta$, the concentration $C$ of the target gas and the local volume $V_{\text{S}}$ of the air near the sensor:

$$N_{\text{S}} = \eta \alpha C V_{\text{S}} \tag{1}$$

where the term

$$\alpha = \left( \frac{\rho N_{\text{A}}}{M_{\text{a}}} \right) \tag{2}$$

is used to convert from the volume of air to the number of air molecules. Here, $\rho$ =1.225 kg m$^{-3}$ is the density of air[1], $N_{\text{A}} = 6.02 \times 10^{23}$ mol$^{-1}$ is Avogadro's constant [2], and $M_{\text{a}} = 28.97$ g mol$^{-1}$ is the molar mass of air [1]. The volume $V_{\text{S}}$ of air contains molecules slow enough to have time to diffuse onto the sensor. It is the product of sensor surface area $A_{\text{S}}$ and the distance $x$ that a molecule can travel via diffusion:

$$V_{\text{S}} = A_{\text{S}} x. \tag{3}$$

The diffusion distance $x$ that can be traveled in a time $t_{\text{S}}$ may be written [1] as

$$x = \sqrt{2 D t_{\text{S}}}. \tag{4}$$

where $D$ is the diffusion coefficient which is equal to $11 \times 10^{-6}$ m$^2$ s$^{-1}$ for ethanol vapor in air. The distance $x$ is drawn in Figure 1b-c.

In Figure 1b the slow-moving molecule is within the distance $x$. It therefore has a greater chance to reach the sensor than the molecule in the higher axial velocity flow of Figure 1c.

The time $t_{\text{S}}$ the target gas has to diffuse onto the sensor may be written as the ratio of the diameter of the sensor $d_{\text{S}}$, and the axial velocity $U_{\text{Z}}$

$$t_{\text{S}} = \frac{d_{\text{S}}}{U_{\text{Z}}}. \tag{5}$$

This is the characteristic time the molecule has before it has drifted past the sensor.

The above theory is perfectly general, and can be applied to any flows where diffusion of molecules onto a sensor is of interest. In Spencer et. al. 2020 [3], we applied this model to compute the molecules diffusing onto an angled moth antenna.

For the case of sniffing, our sensor is inside a circular channel with a radius $R$. In this case, the axial velocity $U_{\text{z}}$ varies across the chamber radius according to the Womersley number and is a function of time $t$, initial phase $\phi$, and the dimensionless distance across the channel $y = r/R$ where $r$ is the radial coordinate and $R$ is the channel radius. The velocity profiles can be found analytically using the process originally outlined by J. R. Womersley in 1955 [4]. Womersley starts with the equation of motion in cylindrical coordinates with a periodic pressure gradient:

$$\frac{\partial^2 U_{\text{z}}}{\partial r^2} + \frac{1}{r}\frac{\partial U_{\text{z}}}{\partial r} - \frac{1}{\nu}\frac{\partial U_{\text{z}}}{\partial t} = -\frac{P_{\text{a}}}{\mu} e^{int} \tag{6}$$

where $\nu$ is the kinematic viscosity of air, $\mu$ is the dynamic viscosity of air, $P_{\text{a}}$ is the applied maximum pressure, and $i$ is the imaginary number $\sqrt{-1}$. The inclusion of $e^{int}$ indicates that the expression is periodic in time with a frequency of $f = n/2\pi$ where $n$ is the angular frequency of the associated waveform in radians per second.

Using the dimensionless distance $y = r/R$, Womersley solves the governing equation of motion, Equation (6), in terms of time $t$, the initial phase $\phi$ and the dimensionless group now attributed to his name, the Womersley number, Wo=$R\sqrt{\frac{2\pi f}{\nu}}$:

$$U_{\text{z}}(y, t, \text{Wo}, \phi) = \frac{P_{\text{a}} R^2}{\mu \text{Wo}^2}\sqrt{1 + J^2 - 2J\cos K} \cdot \sin\left(2\pi f t + \phi + \tan^{-1}\left(\frac{J \sin K}{1 - J\cos K}\right)\right) \tag{7}$$

with J and K given by

$$J = \frac{M_0(y\text{Wo})}{M_0(\text{Wo})}, \tag{8}$$

$$K = \theta_0(\text{Wo}) - \theta_0(y\text{Wo}), \tag{9}$$

and with the functions $M_0$ and $\theta_0$ referring to the modulus and phase of the complex coordinate system respectively. Values for these functions are tabulated by McLachlan [5] for Wo values of up to 45. The respective equations for $M_0$ and $\theta_0$ may be written

$$M_0 = \sqrt{\text{ber}^2 + \text{bei}^2}, \tag{10}$$

$$\theta_0 = \tan^{-1}\left(\frac{\text{bei}}{\text{ber}}\right), \tag{11}$$

where $ber$ and $bei$ are the real and imaginary parts of the first solution to Bessel's equation of order zero. For Wo less than 12, $ber$ and $bei$ can be approximated by a 6 term expanded series,

$$\text{ber}(z) = 1 - \frac{\left(\frac{1}{2}z\right)^4}{2!} + \frac{\left(\frac{1}{2}z\right)^8}{4!} \ldots - \frac{\left(\frac{1}{2}z\right)^{20}}{10!} \tag{12}$$

$$\text{bei}(z) = \left(\frac{1}{2}z\right)^2 - \frac{\left(\frac{1}{2}z\right)^6}{3!} + \frac{\left(\frac{1}{2}z\right)^{10}}{5!} \ldots - \frac{\left(\frac{1}{2}z\right)^{22}}{11!} \tag{13}$$

where $z$ is any real number and varies across the channel cross section from 0 to $y$Wo in this application.

The axial velocity profile $U_{\text{Z}}$ changes with time throughout the phase of the sniffing cycle. A typical velocity profile is shown in Supplementary Figure 1a ($f = 5\,\text{Hz}$, $t = 1/f$, $R = 0.5\,\text{cm}$, $P_{\text{a}} = 275\,\text{Pa}$, $\nu = 1.48 \times 10^{-5}\,\text{m}^2\ \text{s}^{-1}$, $\mu = 1.81 \times 10^{-5}\,\text{kg (m·s)}^{-1}$, $\phi = 0$, Wo= 7.3). Since the flow profile is symmetric around $y = 0$, we only show the top half of the profile. Note $y = 0$ corresponds to the channel mid-line and $y = 1$ to the wall.

Using Equation (5), we use the velocity at each point $y$, to calculate the time $t_{\text{S}} = \frac{d_{\text{S}}}{U_{\text{Z}}(y,t,\text{Wo},\phi)}$ that an odor molecule at that position would take to diffuse to the sensor. We substitute this diffusion time $t_{\text{S}}$ into Equation (4) to obtain the maximum one-dimensional diffusion travel distance $x(t, y) = \sqrt{\frac{2Dd_{\text{S}}}{U_{\text{Z}}(y,t,\text{Wo},\phi)}}$ at each location across the channel's radius. Note that this travel distance $x$ depends on time and position $y$ because it is calculated from the velocity field which varies with time and space. We convert the diffusion travel distance $x$ to dimensionless units by dividing by channel radius $R$ to find $x^* = \frac{1}{R}\sqrt{\frac{2Dd_{\text{S}}}{2U_{\text{Z}}(y,t,\text{Wo},\phi)}}$.

The relationship between dimensionless diffusion distance $x^*$ and position $y$ in the channel is shown by the black curve in Supplementary Figure 1b. The greyed out section indicates positions in the channel where molecules are too far to diffuse onto the sensor. For the 5 Hz example in Supplementary Figure 1b, the closest position where molecules can reach the sensor lies at position $y$ value of 0.85, indicating that at this instant in time, only 15 percent of the channel has opportunities to land on the sensor. The remaining 85 percent of the channel is simply advected away with the flow. At position $y = 0.85$, the velocity is $U_{\text{Z}}$= 2.5 m s$^{-1}$. Using our sensor diameter $d_{\text{S}}$ of 5 mm, the time $t_{\text{S}}$ available for a molecule to diffuse onto the surface is only 2 ms. This time scale represents only 1% of the total sniff cycle time, indicating that they diffuse nearly instantaneously onto the sensor.

The maximum distance $x_{\text{max}}$ that a molecule may diffuse onto the wall is dictated by the intersection of the dimensionless diffusion distance $x^*(t, y)$ and the equation $x^* = 1-y$. As shown in Supplementary Figure 1b, all molecules with positions $y$ greater than $x_{\text{max}}$ may diffuse onto the wall. The equation to solve for the position $y = x_{\text{max}}$ may be written

$$\frac{1}{R}\sqrt{\frac{2Dd_{\text{S}}}{2U_{\text{Z}}(y, t, \text{Wo}, \phi)}} = 1 - y \qquad (14)$$

where the equation for $U_{\text{Z}}$ is given in Equation (7), and the equation is solved numerically using Matlab.

As the velocity profile changes throughout the sniff cycle, so does the distance $x_{\text{max}}$. The function $x_{\text{max}} = x_{\text{max}}(t, \text{Wo}, \phi)$ thus varies continuously and periodically. As an example, we

use MATLAB to compute $x_{\text{max}}$ for frequencies of 1 Hz and 5 Hz, producing the blue and black lines, respectively, in Supplementary Figure 1c. The resulting $x_{\text{max}}$ values are then integrated with respect to time to determine $\int_0^T x_{\text{max}}(t, \text{Wo}, \phi)\, dt$, the total travel distance molecules can diffuse per cycle. This integration is performed numerically using the trapezoidal method. The resulting diffusion travel distance is then substituted into Equation (3) to find the total volume of air $V_{\text{s}}$ with a chance of hitting the sensor via diffusion in each cycle. Similar to the approach in Equation (1), we multiply the resulting volume $V_{\text{s}}$ by the factor $\alpha$ to find the total number of molecules available to reach the sensor via diffusion $N_{\text{d}}$ in a time $T$ cycle. This number of molecules may be written:

$$N_{\text{d}}(T) = \alpha A_{\text{s}} \int_0^T x_{\text{max}}(t, \text{Wo}, \phi)\, dt \tag{15}$$

where the values $x_{\text{max}}$ are found using the method discussed earlier. This number of molecules $N_{\text{d}}$ is not the number of molecules that actually land on the sensor, but the number of molecules that have the potential to do so in a time $T$. It is thus an upper bound for sensing. For simplicity, we refer to them as the number of available molecules for collection.

If experiments are done with a collection efficiency $\eta$ and concentration of target gas $C$, the number of molecules arriving in a time $T$ cycle may be written $N_{\text{s}}(T) = \eta C N_{\text{d}}(T)$. However, multiplying by $\eta$ and $C$ does not change the trends found in Supplementary Figure 1d but would only serve to scale the final values. Thus, from hereon we simply neglect consideration of the efficiency $\eta$. Since the sensitivity of the sensor to the ethanol is unknown, we use a single fitting factor $\beta = 4.6$ mA per ethanol molecule to produce the equation:

$$A = \beta C N_{\text{d}} \tag{16}$$

where $A$ is the amplitude of the signal response oscillations in mA. Equation (16) and Equation (15) are the culmination of this model. The amplitude $A$ represents the predicted responses of our sensor as a function of Womerseley number and ethanol concentration.

**Molecules sensed per unit time and unit sniff** The areas under the curves in Supplementary Figure 1c indicate the total region above the sensor for which an odor molecule can reach the sensor surface via diffusion. For illustration, we consider two sniffing frequencies, 5 Hz and 1 Hz, shown by the solid black and dashed blue lines respectively. For both frequencies, the peak $x_{\text{max}}$ values are associated at the transitions between inhalation and exhalation. At these inflection points, the velocity of the air slows down sufficiently that molecules can travel further via diffusion. Conversely, when the air velocity is at a maximum, the diffusion distance $x_{\text{max}}$ is at a minimum. For the $R = 0.5$ cm example simulation of Supplementary Figure 1, the molecules in the 1 Hz flow only have a chance to reach the sensor if they originate from a distance of 3.5 percent of the radius. Thus the air that is smelled is truly a thin ”skin” around the sensor.

Because of its shorter period, the 5 Hz signal in crossed hatched black has a smaller per cycle integral than the 1 Hz signal in horizontal hatched blue in Supplementary Figure 1c. The integral

per cycle is shown by the black anbd blue shaded regions under the curves. This smaller integral is associated with a lower number of available molecules. By computation, we show that as Wo increases, the data from a single sniff decreases exponentially, as shown by the red dashed line of Supplementary Figure 1d. This may explain why animals sniff in such a small range of Womersley numbers: to avoid the loss of data per sniff. Conversely, to maximize the data per sniff, it is better to sniff slower, and thus at slower Wo number.

Now we consider the information gathered per unit time. The molecules available for collection per unit time was found by taking the molecules available for collection per cycle and multiplying by the number of cycles per second. In Supplementary Figure 1c with a time scale of 1 second, the area under the 5 Hz signal in cross hatched black exceeds the area under the 1 Hz signal in horizontal hatched blue. This is due to the 5 Hz signal having more peaks and a lower maximum velocity. The total number of molecules per second appears to increase linearly as shown by the solid green line of Supplementary Figure 1d. The inverse relationship between the dashed red and solid green curves of Supplementary Figure 1d visually illustrates the trade-off between the amount of information gleaned per cycle in red and per unit time in green.

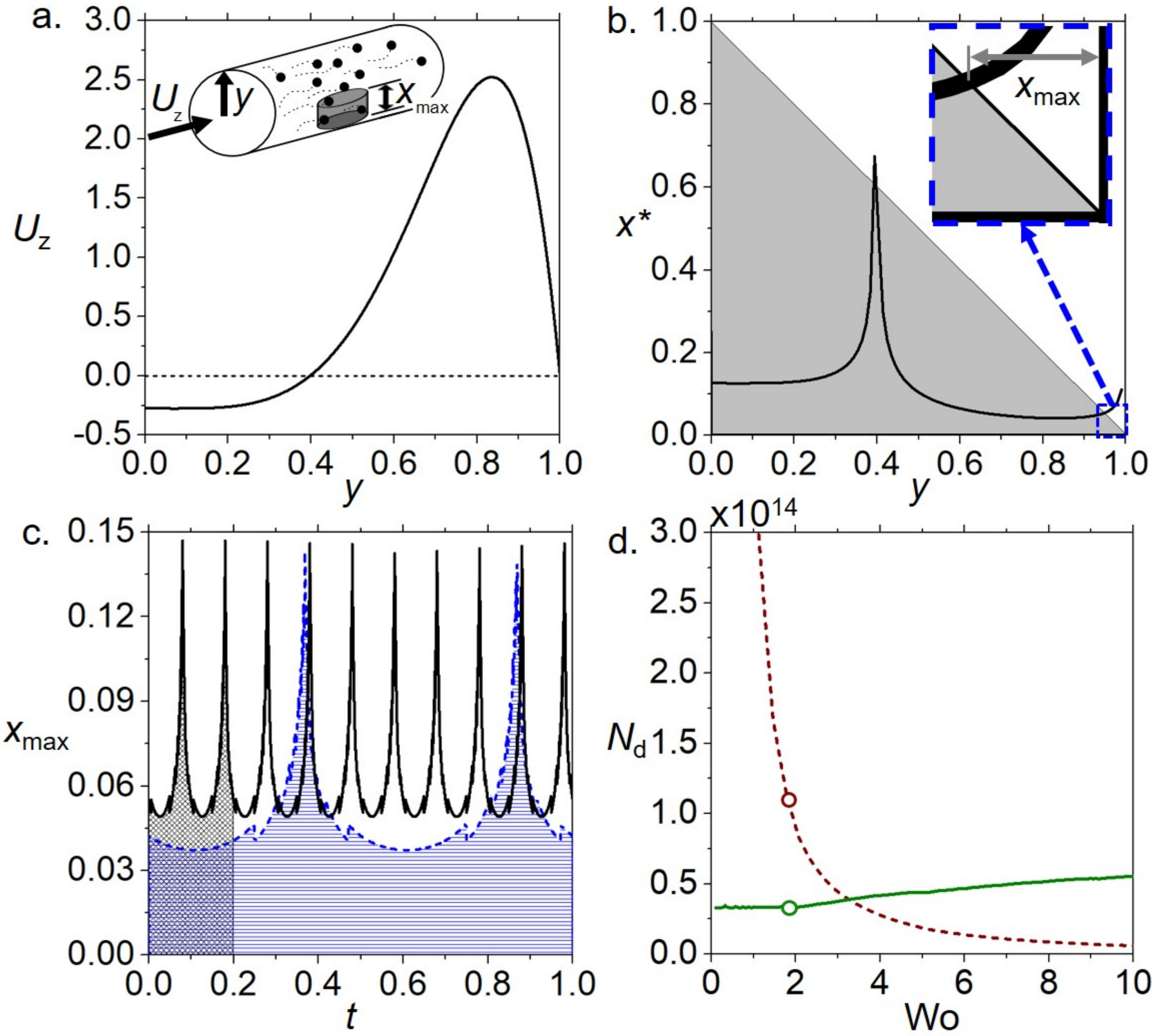


**Supplementary Figure 1.** Mathematical methods to calculate molecules arriving on the sensor. **a.** Instantaneous axial velocity $U_Z$ across dimensionless radial distance $y = r/R$ for a sniffing frequency $f$ of 5 Hz. For this and all sub-figures, we use time $t$ of $1/f$, radius $R$ of 0.5 cm, maximum applied pressure $P_a$ of 275 Pa, dynamic viscosity of air $\mu = 1.81 \times 10^{-5}$ kg (m·s)$^{-1}$ , zero initial phase $\phi$, and corresponding Wo of 7.3. **b.** Dimensionless distance in space a molecule could travel via diffusion $x^* = x/R$ at each location $y$. The greyed out section indicates region where a molecule would have no chance of diffusing onto the channel wall. Detail view illustrates the maximum distance $x_{max}$ from the wall that a molecule could reach the edge via diffusion. Visual representation of $x_{max}$ is shown in panel a. **c.** Maximum diffusion distance $x_{max}$ for each instance in time $t$ in seconds of the sniff cycles for frequencies of 1 and 5 Hz in dashed blue and solid black, respectively. Shaded regions represent integral of one sniff cycle consisting of an inhale and exhale. **d.** The relationship between number of available molecules for collection $N_d$ and Womersley number. Red line indicates number of molecules available per cycle. Green line indicates the number of molecules available per second. Open circles represent the optimal collection rates for the 8.9 parts per thousand concentration tests with the chosen sensors (duplicated from main text Figure 6d).

**Supplementary Table 1.** Body mass and sniffing frequency raw data[6–12] used in main text Fig. 1. Measurements of rat, horse, and giraffe frequencies were using YouTube videos by users Angela, FreeAnimalVideo.org, and ZSL London Zoo

| **Animal** | **Body Mass (kg)** | **Max sniffing Frequency (Hz)** | **Reference** |
|---|---|---|---|
| Mouse (male C57BL/6) | 0.02 | 12 | Wesson, 2008 |
| Shrew (*Tupaia belangeri*) | 0.125 | 14 | Holst, 1976 |
| Long-Evans rat | 0.35 | 8 | Youngentob, 1987 |
| Wistar rat | 0.4 | 8.6-14 | Khan, 2012 |
| Long-Evans rat | 0.45 | 6.5 | Wesson, 2009 |
| NZW rabbit | 4.78 | 8 | Xi, 2016 |
| Pomeranian | 6.8 | 6 | Craven, 2009 |
| Beagle | 13.6 | 4.5 | Craven, 2009 |
| Shelti-husky mix | 14.5 | 4.5 | Craven, 2009 |
| Boarder collie-lab mix | 16.8 | 5 | Craven, 2009 |
| German shepherd | 34.5 | 5 | Craven, 2009 |
| Labrador retriever | 37 | 5 | Craven, 2009 |
| Labrador retriever | 52.9 | 6 | Craven, 2009 |
| Horse | 454 | 2.3 | Measured by authors |
| Giraffe | 1179.34 | 1.6 | Measured by authors |
| Elephant | 2300 | 2 | Measured by authors |

**Supplementary References**


1. Cussler, E. L. *Diffusion: mass transfer in fluid systems* (Cambridge University Press, 2009).

2. Deslattes, R. *et al.* Determination of the Avogadro constant. *Phys. Rev. Lett.* **33**, 463 (1974).

3. Spencer, T. L. *et al.* Moth-inspired methods for particle capture on a cylinder. *Journal of Fluid Mechanics* **884** (2020).

4. Womersley, J. R. Method for the calculation of velocity, rate of flow and viscous drag in arteries when the pressure gradient is known. *J. Physiol.* **127**, 553–563 (1955).

5. McLachlan, N. W. Bessel functions for engineers (1955).

6. Wesson, D. W., Donahou, T. N., Johnson, M. O. & Wachowiak, M. Sniffing behavior of mice during performance in odor-guided tasks. *Chem. Senses* **33**, 581–596 (2008).

7. Holst, D. v. & Kolb, H. Sniffing frequency oftupaia belangeri: A measure of central nervous activity (arousal). *J. Comp. Physiol.* **105**, 243–257 (1976).

8. Youngentob, S. L., Mozell, M. M., Sheehe, P. R. & Hornung, D. E. A quantitative analysis of sniffing strategies in rats performing odor detection tasks. *Physiol. Behav.* **41**, 59–69 (1987).

9. Khan, A. G., Sarangi, M. & Bhalla, U. S. Rats track odour trails accurately using a multi-layered strategy with near-optimal sampling. *Nat. Commun.* **3**, 703 (2012).

10. Wesson, D. W., Verhagen, J. V. & Wachowiak, M. Why sniff fast? the relationship between sniff frequency, odor discrimination, and receptor neuron activation in the rat. *J. Neurophysiol.* **101**, 1089–1102 (2009).

11. Xi, J. *et al.* Anatomical details of the rabbit nasal passages and their implications in breathing, air conditioning, and olfaction. *Anat. Rec.* **299**, 853–868 (2016).

12. Craven, B. A., Paterson, E. G., Settles, G. S. & Lawson, M. J. Development and verification of a high-fidelity computational fluid dynamics model of canine nasal airflow. *J. Biomech. Eng.* **131**, 091002 (2009).
